# rs1495741 as a tag single nucleotide polymorphism of N-acetyltransferase 2 acetylator phenotype associates bladder cancer risk and interacts with smoking: A systematic review and meta-analysis

**DOI:** 10.1097/MD.0000000000004417

**Published:** 2016-08-07

**Authors:** Chong Ma, Liyan Gu, Mingyuan Yang, Zhensheng Zhang, Shuxiong Zeng, Ruixiang Song, Chuanliang Xu, Yinghao Sun

**Affiliations:** a Department of Urology; b Department of Nursing; c Department of Spine Surgery, Changhai Hospital, Second Military Medical University, Shanghai, China.

**Keywords:** bladder cancer, meta-analysis, NAT2, rs1495741, smoking

## Abstract

Rs1495741 has been identified to infer N-acetyltransferase 2 (*NAT2*) acetylator phenotype, and to decrease the risk of bladder cancer. However, a number of studies conducted in various regions showed controversial results. To quantify the association between rs1495741 and the risk of bladder cancer and to estimate the interaction effect of this genetic variant with smoking, we performed a systematic literature review and meta-analysis involving 14,815 cases and 58,282 controls from 29 studies. Our results indicates rs1495741 significantly associated with bladder cancer risk (OR = 0.85, 95% CI = 0.82–0.89, test for heterogeneity *P* = 0.36, *I*^2^ = 7.0%). And we verified this association in populations from Europe, America, and Asia. Further, our stratified meta-analysis showed rs1495741's role is typically evident only in ever smokers, which suggests its interaction with smoking. This study may provide new insight into gene-environment study on bladder cancer.

## Abstract 2

Supplemental Digital Content is available in the text

## 1 Introduction

Bladder cancer is a common malignancy internationally, with >357,000 new cases per year.^[1]^ For its poor prognosis of muscle-invasive bladder cancer, early identification and intervention for high-risk population is of significance.^[2]^ Genetic variants, environmental factors, and their interaction have been confirmed to participate in bladder cancer development.^[3]^

N-acetyltransferase 2 (NAT2) could influence the detoxification of chemical carcinogens including aromatic amines, the most important one for bladder cancer in tobacco.^[4,5]^ Generally, *NAT2* phenotype can be classified into slow, intermediate, and rapid acetylator.^[3]^ By inferring *NAT2* phenotype with combinations of single nucleotide polymorphisms (SNP), previous meta-analyses have associated NAT2 slow acetylator phenotype with bladder cancer risk.^[6,7]^ Recently, a novel SNP located in 8p22, rs1495741 G/A, was identified to correlate with bladder cancer risk.^[8,9]^ Rs1495741 locates ∼10 kb from 3′ of *NAT2* and could predict *NAT2* phenotype with high accuracy.^[8]^ Plausibly, this association with bladder cancer was reported to be limited to smokers in some studies.^[9,10]^ However, multiple studies on this relationship in different regions and designs turned out with different results.^[9–12]^ To comprehensively estimate the association of *NAT2* tag SNP (rs1495741) and smoking with bladder cancer, we conducted this systematic review and meta-analysis.

## 2 Methods and materials

This systematic review and meta-analysis was conducted under the guidance of Meta-analysis of Observational Studies in Epidemiology (MOOSE).^[13]^

Ethical approval is not required for this meta-analysis since data were extracted from previous published studies.

### 2.1 Search strategy

A comprehensive online search was conducted on PubMed, Embase, Web of Science, WanFang Data from the earliest date to September 28, 2015. Free-text words (“bladder cancer or urothelial carcinoma”) and (“N-acetyltransferase 2” OR “NAT2”) and (“single nucleotide polymorphism” OR “genetic variation” OR “rs1495741”) were searched, with no language or other restrictions. Then studies investigating the association between rs1495741, smoking, and bladder cancer risk were selected through the title and abstract. The whole text was reviewed if information in the title and abstract is not sufficient to make a decision. Secondary searches of literature were conducted by searching the reference lists of the selected studies and relevant reviews to avoid missing.

### 2.2 Inclusion/exclusion criteria

Case-control or prospective cohort studies were included in our meta-analysis. Patients diagnosed with bladder cancer confirmed by pathology were included in the case group. Reviews, correspondences, editorial articles, and meeting reports were excluded.

### 2.3 Data extraction

Two investigators independently reviewed and extracted data from all the eligible publications. Disagreement was resolved by consensus. Studies in different regions and on different populations were considered as individual ones. As data from several studies has been published more than once in the included articles, we extracted data only from the updated articles to enlarge sample size and avoid duplication. The following data were extracted: study name, study region, year of publication, study design (case-control or cohort study), number of cases and controls, smoking status, and allele distribution of rs1495741. AA, GA, GG genotype of rs1495741 was assumed to represent slow, intermediate, and rapid phenotype of NAT2, respectively.^[8,14]^ For articles with deficient information of alleles and genotypes to calculate the odds ratios (ORs), we tried to obtain data from their authors. Smoking status was categorized into ever smoker (former and current smoker combined) and never smoker. As the descriptions of “ever smoker,” “never smoker,” “former smoker,” and “current smoker” were different in relevant studies and could not be recategorized under the same standard, we classified each subject according to their previous study.

### 2.4 Quality assessment

SNP in all included studies was in Hardy–Weinberg equilibrium (HWE). We used chi squared distribution to calculate HWE and the corresponding *P* values were given in Supplemental Table 1. As there is no widely accepted criterion to evaluate the quality of SNP-related studies, like many of the previous articles, we did not consider parameters other than HWE.^[3,6]^

### 2.5 Statistical analysis

The association strength of rs1495741 and bladder cancer risk was measured by odds ratio (OR) with 95% CI. In this study, we referred to allele G to calculate ORs. If the data was adequate, 3 classical genetic modes: dominant models ([GG + GA] vs AA), recessive model (GG vs [GA + AA]), and additive mode (total G allele vs total A allele) were used to detect the association.^[15]^ In addition, GG versus AA and GG versus GA models were also calculated. To analyze the ethnic and local environmental factors combined, we conducted subgroup analysis by study region. And to identify the difference between study designs, subgroup analysis was also performed. The association between bladder cancer and smoking status of the included populations were first estimated by pooled OR. Then the interaction of rs1495741 and smoking was investigated by stratified analysis by smoking habits (ever and never smoking groups). Underlying inter-study heterogeneity was measured using *Q* test and quantified by *I*^2^, defining *P* value <0.05 and *I*^2^ more than 50% as significant heterogeneity.^[16]^ The fixed effects model was used to pool ORs and 95% confidential interval (CI) when there was no significant heterogeneity; otherwise, the random effects model was applied. Sensitivity analyses were performed to assess the stability of obtained pooled ORs by omitting a single article each time to test its influence on the pooled results. And we used Begg's and Egger's method to test publication bias. All statistical analyses were conducted using Stata 12.0 software (StataCorp, College Station, TX).

## 3 Results

### 3.1 Study characteristics

**Figure 1:**
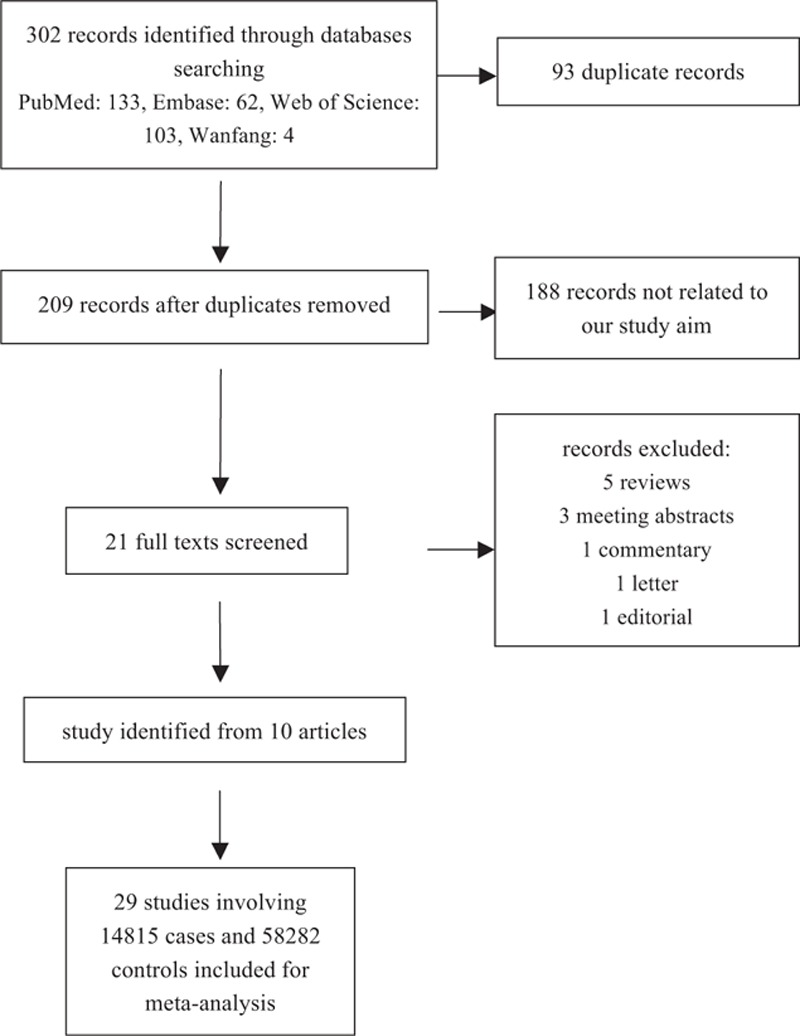
Flowchart for the study selection process.

A total of 302 records, 133 from Pubmed, 62 from Embase, 103 from Web of Science and 4 from Wanfang Database were found under our search strategy. The complete literature selection process was shown in Fig. 1. By reviewing title and abstract, we excluded 281 articles, including 93 duplicate ones and 188 articles irrelevant to our objective. After full text screening, we removed other 11 records:5 reviews,^[5,17–20]^ 3 meeting abstracts,^[21–23]^ 1 letter to editor,^[24]^ 1 commentary,^[25]^ 1 editorial.^[26]^ After data extraction from the left 10 articles,^[8–12,14,27–30]^ 29 individual studies with 14,815 cases and 58,282 controls were enrolled in the present study to estimate relation of rs1495741 and bladder cancer risk. Of the included study, 14 studies were conducted in Europe, 13 in America (12 in USA and 1 in Venezuela, Latin America), and 2 in Asia. As regards to study design, 22 were retrospective case-control studies (10 hospital-based, 4 population-based, and 8 without further description) and 7 were prospective cohort studies. Lutherstadt Wittenberg Bladder Cancer Study (LWBCS) in Rothman et al's article was named East Germany Study (East Germany) in Selinski et al's publication on the same population.^[9,11]^ We only included the data from Selinski et al's article to avoid duplication. The general characteristics of each study included in this meta-analysis were summarized in Table 1. We calculated pooled OR of other inheritance models based on genotype distribution data from 8 studies, whose details were shown in Supplemental Table 1.

**Table 1:**
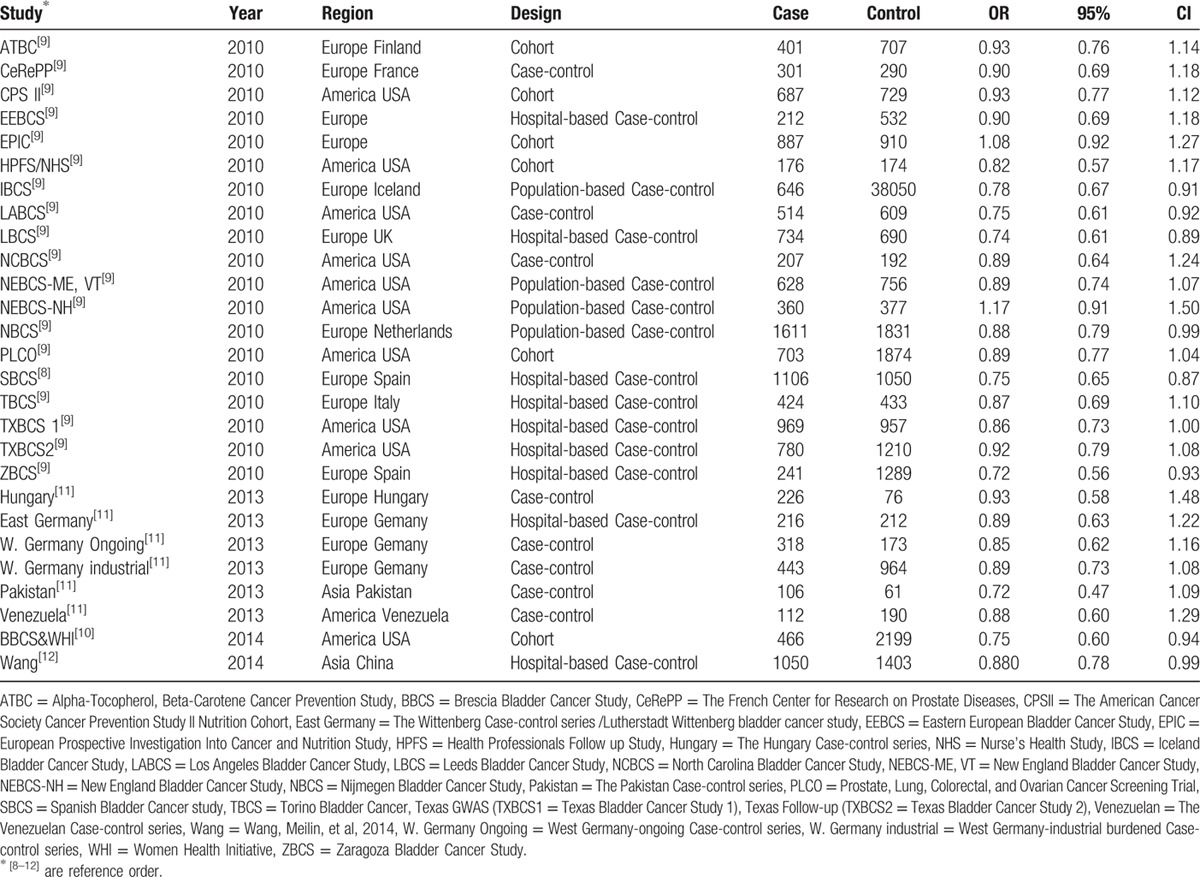
Characteristics of the included studies.

To assess smoking risk, data from 25 of the 29 included studies were available for pooling OR. The interaction of rs1495741 and smoking were investigated by rs1495741 AA (NAT2 slow acetylator) compared to GG/GA (NAT2 intermediate/rapid acetylators) stratified by smoking status (ever and never smoker) based on 24 individual studies.

## 4 Meta-analysis results

### 4.1 rs1495741

**Figure 2:**
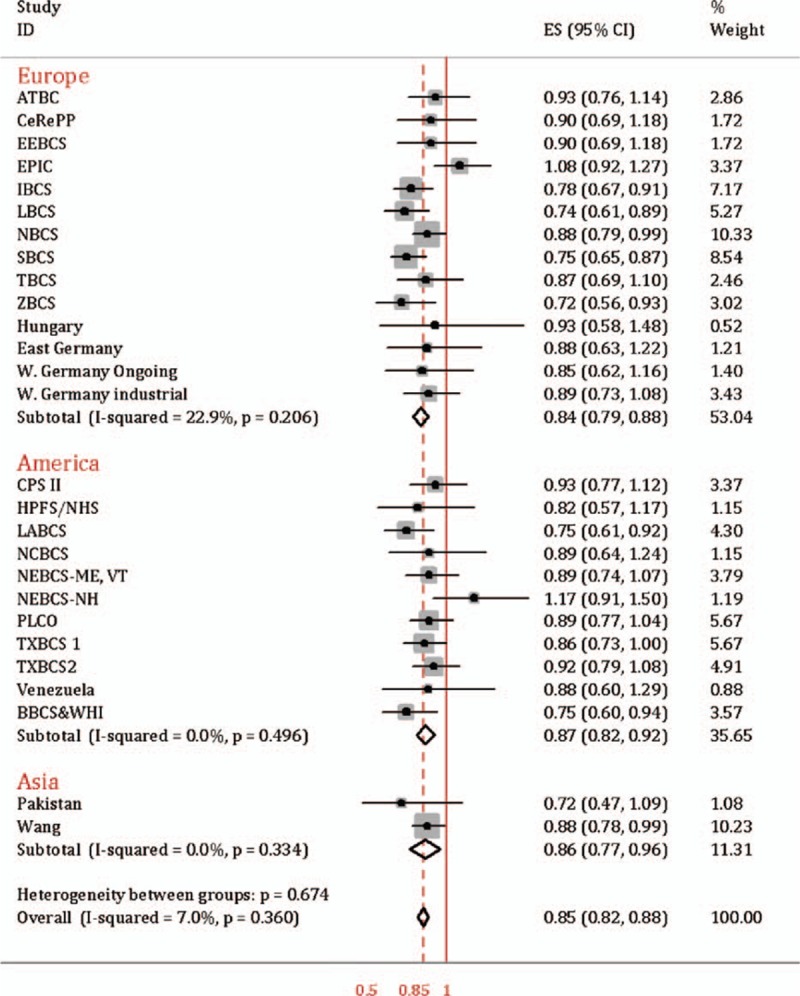
rs1495741 and the risk of bladder cancer in different study regions.

**Figure 3:**
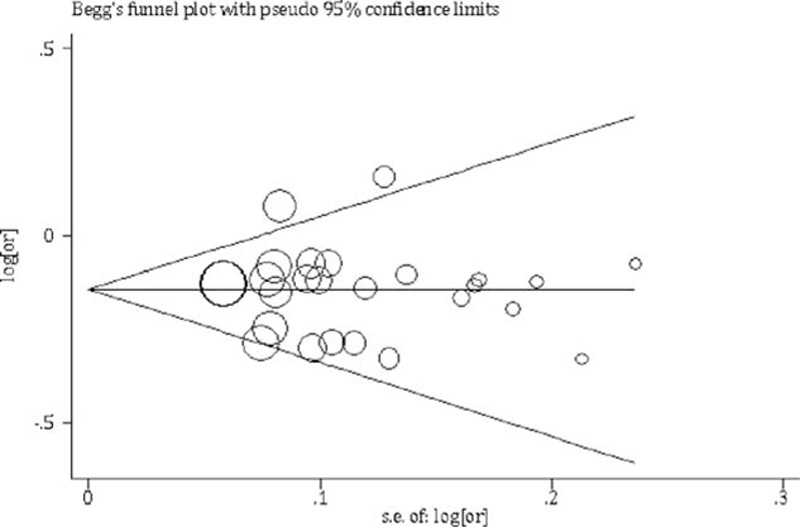
Funnel graph of publications of rs1495741 and bladder cancer.

As regards to G allele, rs149574 significantly reduced susceptibility to bladder cancer (OR = 0.85, 95% CI = 0.82–0.88, test for heterogeneity *P* = 0.36, *I*^2^ = 7.0%). Subgroup analysis by study region verified this association with OR = 0.84, 95% CI = 0.79–0.88 in Europe, OR = 0.87, 95% CI = 0.82–0.92 in America and OR = 0.86, 95% CI = 0.77–0.96 in Asia respectively and heterogeneity between groups *P* = 0.67 (Fig. 2). As for different study design (Supplemental Figure 1), rs1495741 was significantly negative associated with bladder cancer risk in cohort study (OR = 0.91, 95% CI = 0.83–0.98), hospital-based case-control study (OR = 0.83, 95% CI = 0.78–0.88), population-based case-control study (OR = 0.87, 95% CI = 0.80–0.93), and case-control study without further description (OR = 0.83, 95% CI = 0.75–0.92). Sensitivity analysis showed that the association between rs1495741 and bladder cancer was solid when any single study was omitted (Supplemental Figure 2). Both Begg's (*P* = 0.49) and Egger's test (*P* = 0.84) identified no significant publication bias, as can be seen in funnel graph (Fig. 3).

The meta-analysis results of other inheritance models were based on 8 available studies: dominant mode ([GG + GA] vs AA) OR = 0.80, 95% CI = 0.72 to 0.88; recessive mode GG versus (GA+AA) OR = 0.81, 95% CI = 0.70 to 0.94; GG versus AA OR = 0.78, 95% CI = 0.66 to 0.93; GG versus GA OR = 0.86, 95% CI = 0.74 to 1.01. The corresponding forest plots and heterogeneity test results were represented in Supplemental Figure 3 to Supplemental Figure 6.

### 4.2 Smoking

Based on meta-analysis of 25 studies from the included ones, pooled OR of ever smoker versus never smoker (OR = 2.20, 95% CI = 1.80–2.68, heterogeneity *P* = 0.00, *I*^2^ = 92.0%, by the random effect method) confirmed smoking as a strong risk factor of bladder cancer. Subgroup analysis by region showed OR = 2.71, 95% CI = 2.30 to 3.20 in Europe, OR = 2.098, 95% CI = 1.62 to 2.72 in America and OR = 0.59, 95% CI = 0.26 to 1.36 in Asia (Supplemental Figure 7). As regard to study design, we observed different association strengths between smoking and bladder cancer in cohort study (OR = 1.48, 95% CI = 1.02–2.14), hospital-based case-control study (OR = 2.35, 95% CI = 1.51–3.65), population-based case-control study (OR = 2.79, 95% CI = 2.33–3.34), and case-control study without further description (OR = 2.38, 95% CI = 1.69–3.34), as can been seen in Supplemental Figure 8.

### 4.3 rs1495741–smoking interaction

**Figure 4:**
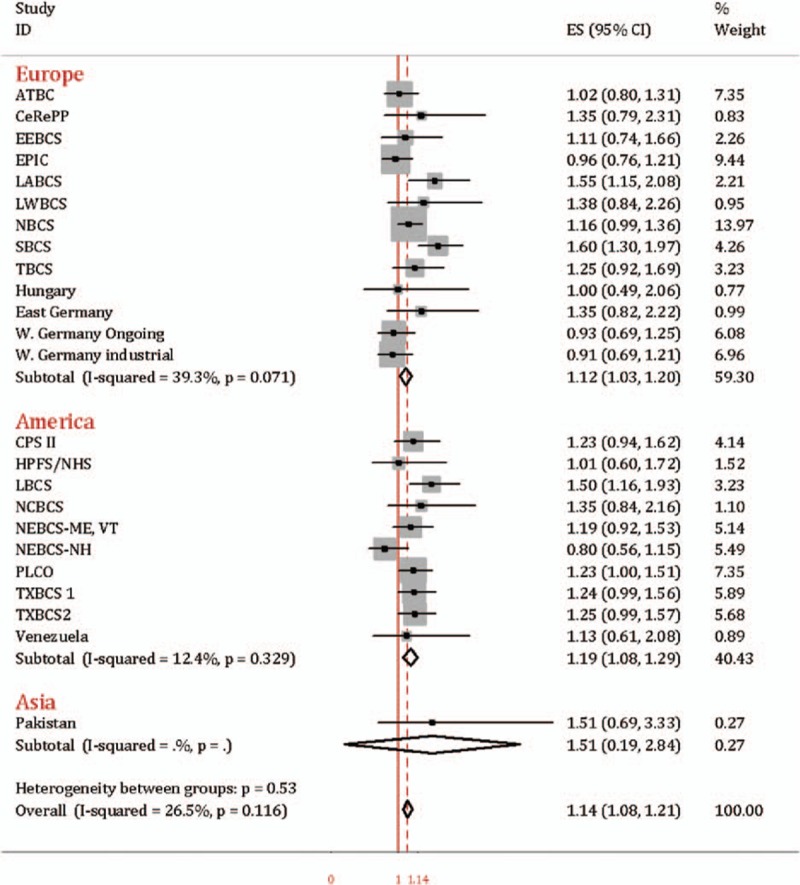
Forest plot of rs1495741 AA vs. GG/GA in ever smoker.

**Figure 5:**
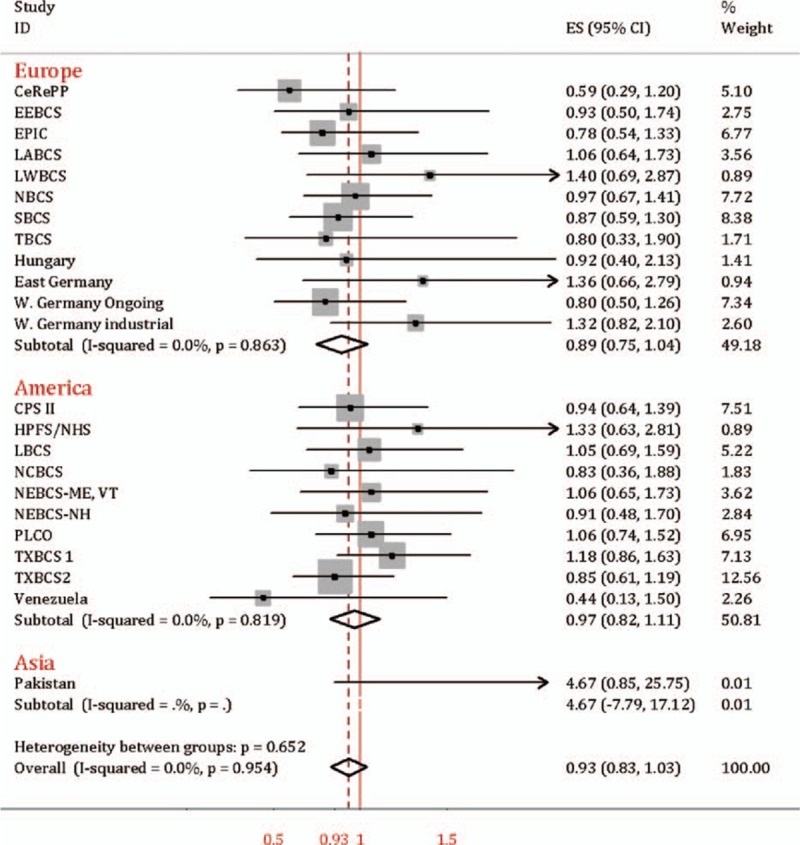
Forest plot of rs1495741 AA vs. GG/GA in never smoker.

Compared to rs1495741 GG and GA genotypes (*NAT2* intermediate and rapid acetylation phenotypes), the association between rs1495741 AA genotype (*NAT2* slow acetylator) and bladder cancer susceptibility was evident in ever smoker (OR = 1.14, 95% CI = 1.08–1.21). And this result was consistent with subgroup analysis in Europe (OR = 1.12, 95% CI = 1.03–1.21) and America (OR = 1.19, 95% CI = 1.08–1.29), as was presented in Fig. 4. However, in nonsmoker, rs1495741 AA was not significantly increased susceptibility to bladder cancer when compared to GG and GA genotypes combined (OR = 0.93, 95% CI = 0.83–1.03, test for heterogeneity *P* = 0.95, *I*^2^ = 0.0%), shown in Fig. 5. Only the data from Pakistan study was from Asian population, which has been published before.^[11]^

## 5 Discussion

In this study, we included the largest population ever to estimate the association of SNP rs1495741, smoking, and bladder cancer risk. By updating the prior results from the genome-wide association study (GWAS) with additional data from Europe and Asia, we confirmed that SNP rs1495741 G/A is significantly associated with susceptibility of bladder cancer. And through stratified meta-analysis of rs1495741–smoking interaction, we verified this association is typically evident in ever smoker population. These results are consistent with rs1495741's role as a tag SNP of *NAT2* acetylation phenotypes. Small inter-study heterogeneity showed in *Q* test and *I*^2^ and insignificant publication bias suggested the power of our results.

Differing from the previous study on *NAT2*, this study focused on *NAT2* tag SNP rs1495741 and its genotypes on bladder cancer risk.^[3]^ Garcia-Closas et al identified and Rothman et al and Figueroa et al validated the association between rs1495741 and the risk of bladder cancer in Caucasians from USA and some regions of Europe.^[8–10]^ Prior articles also reported the interaction of rs1495741 and smoking.^[9,10]^ Nevertheless, these results were not evident by the following studies in Asians and Caucasians from other regions of Europe.^[11,27]^ This inconsistency could result from relatively small sample size, from genetic ethnical heterogeneity or from unknown local environmental factors other than smoking.^[31,32]^ Selinski et al^[11]^ explained their results by an extra “ultra-slow” genotype of NAT2. Our subgroup analysis by region entailed ethnical and local factors together and confirmed the relationship of rs1495741 and bladder cancer in ethnic groups of Asians and Caucasians including other regions of Europe. In our subgroup analysis of study design, we also verified this connection in either prospective or retrospective study. As rs1495741 has also been used to predict NAT2 acetylator phenotype in the studies of diabetes, colorectal cancer, and personalized medication, our results could add information to the understanding of rs1495741.^[32–34]^

To estimate the interaction of rs1495741 and smoking, we first confirmed the adverse effect of smoking in our enrolled population. As the data of smoking status was obtained by questionnaire, we tried to minimize recall bias and differential misclassification by subgroup analysis of prospective or retrospective study design. Prospective cohort study turned out with lower OR in this study, which could result from the decrease of confounders and from the different definitions of “never smoker” in the included studies. PLCO, EPIC, CeRePP, and so on, defined never smoker as subjects who smoked <6 months in their lifetime, whereas that of SBCS, NEBCS, and so on, were subjects had smoked <100 cigarettes over their lifetime.^[10]^ Notably, stratified meta-analysis by smoking status for rs1495741 AA (NAT2 slow acetylator) compared to GG/GA (NAT2 intermediate/rapid acetylators) implies that rs1495741 associates the risk of bladder cancer through mechanisms of NAT2 detoxification of carcinogens in tobacco. Our results epidemiologically confirmed NAT2's role in the development of bladder cancer, which was reported by prior articles.^[3,9]^ Figueroa and his colleagues have identified this interaction associated with smoking intensity and smoking duration.^[7]^

There are some limitations in this study. First, the relevant studies for meta-analysis had differences in the method to obtain OR. Logistic regression models were adjusted by different combinations of confounders, such as, age, sex, region, and smoking status, which may generate potential bias in our study. Second, definitions of “ever smoker” and “never smoker” were not consistent in each study. We categorized each subject as their previous definition in each corresponding reference. With more data becoming available, we might recategorize the population under the same standard. Third, sample size from Asia was smaller than that from Europe and America. More studies on rs1495741 and bladder cancer for Asian might make our results more convincing in the future.

## 6 Conclusions

Our systematic review and meta-analysis indicates the association of SNP rs1495741, smoking, and bladder cancer risk in populations from Europe, America, and Asia. And we confirmed this association in ever smoker population only, which suggests the underlying mechanism of this association could be rs1495741's role as a tag SNP of NAT2 acetylation phenotype. Our results may provide new insights into gene-environmental study on bladder cancer.

## Supplementary Material

Supplemental Digital Content
