# Supplementary material for: rs1495741 as a tag single nucleotide polymorphism of N-acetyltransferase 2 acetylator phenotype associates bladder cancer risk and interacts with smoking: A systematic review and meta-analysis
Source: Medicine (Baltimore). 2016 Aug 7;95(31):e4417. doi: 10.1097/MD.0000000000004417 (PMC4979814; doi:10.1097/MD.0000000000004417)
Supplement: Supplemental Digital Content [file medi-95-e4417-s001.doc]

Supplemental Figure 1. Rs1495741 and the risk of bladder cancer in different study designs


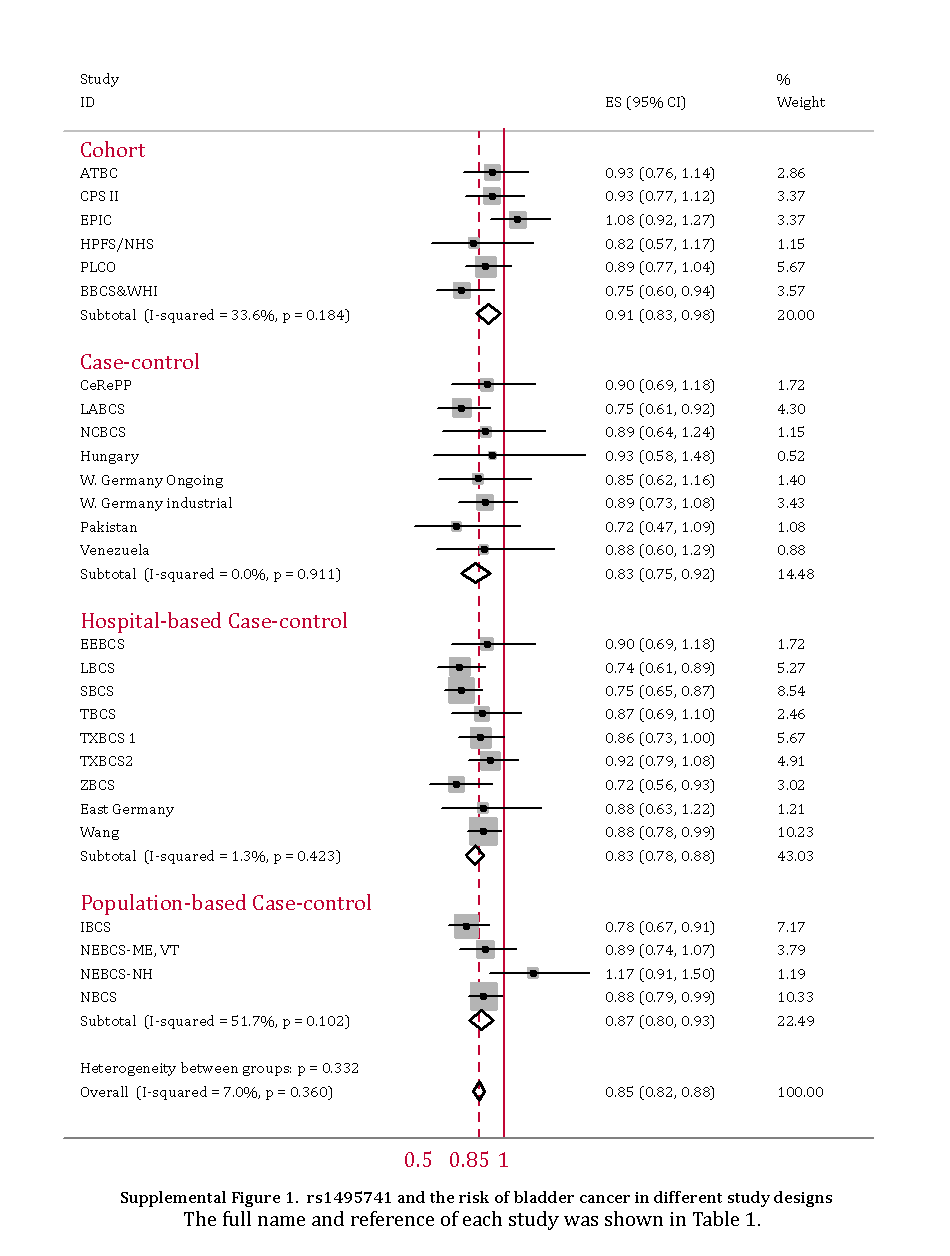


Supplemental Figure 2. Sensitivity analysis of rs1495741 and bladder cancer risk


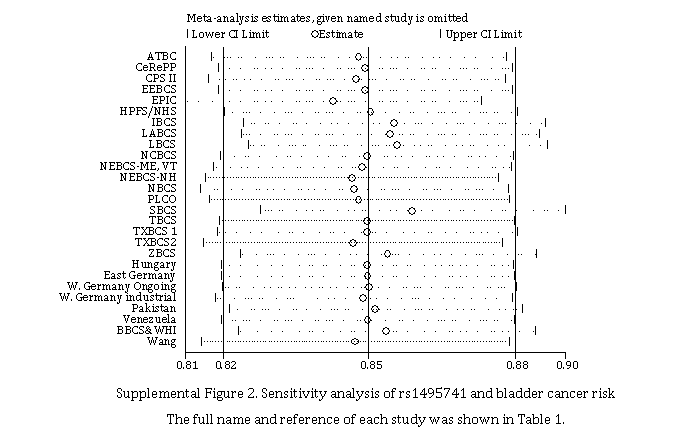


Supplemental Figure 3. rs1495741 GG vs. AA model and the risk of bladder cancer


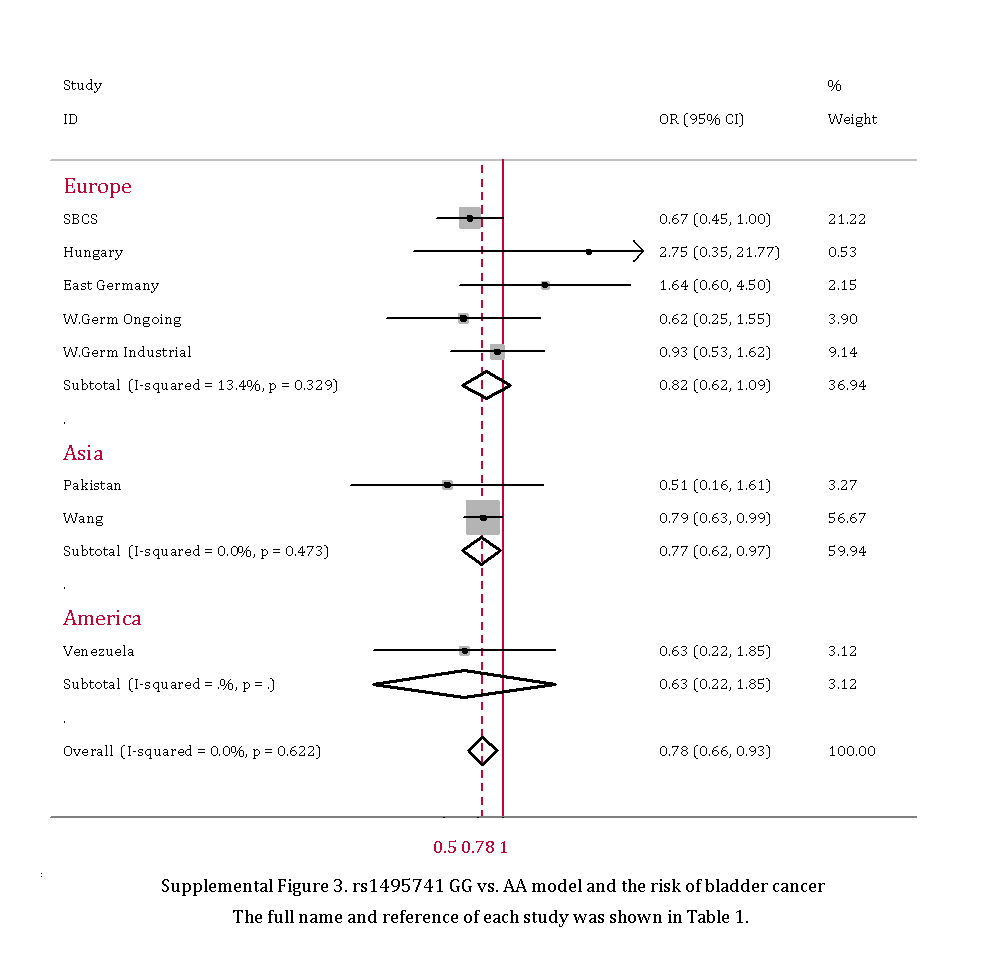


Supplemental Figure 4. rs1495741 GG vs. GA model and the risk of bladder cancer


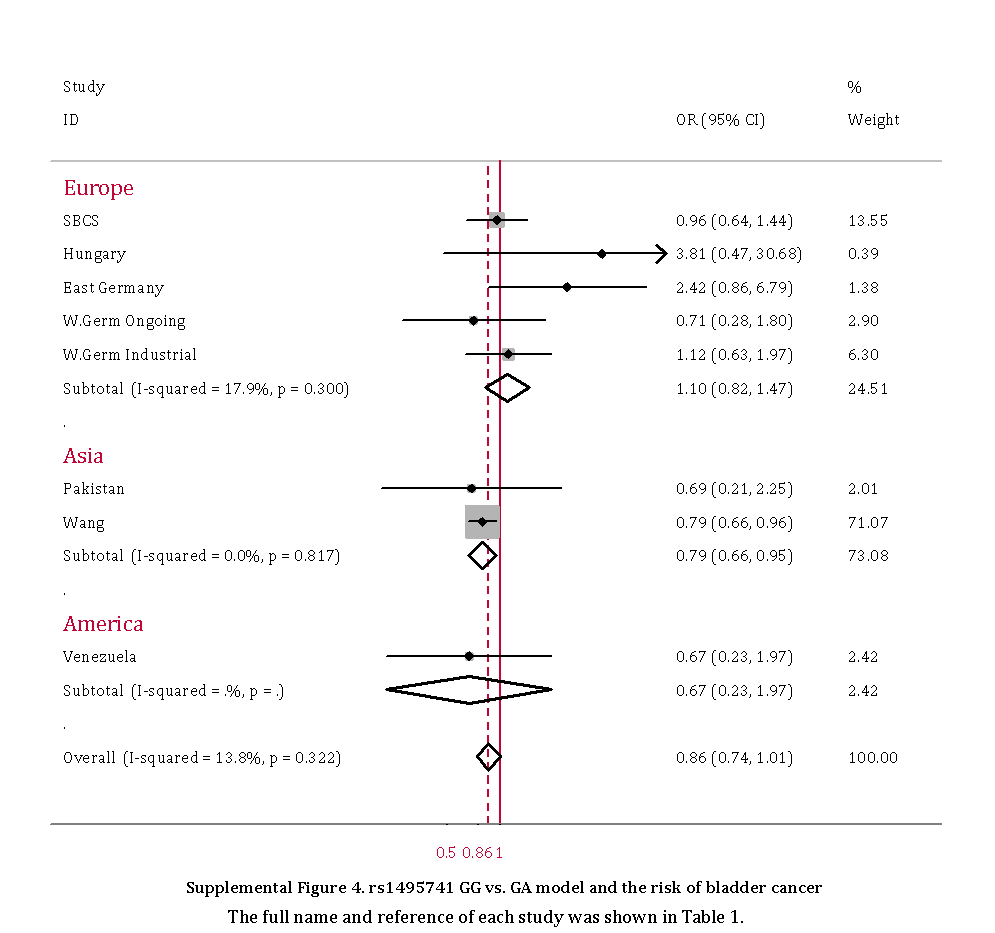


Supplemental Figure 5. rs1495741 GG vs. (GA+AA) model and the risk of bladder cancer


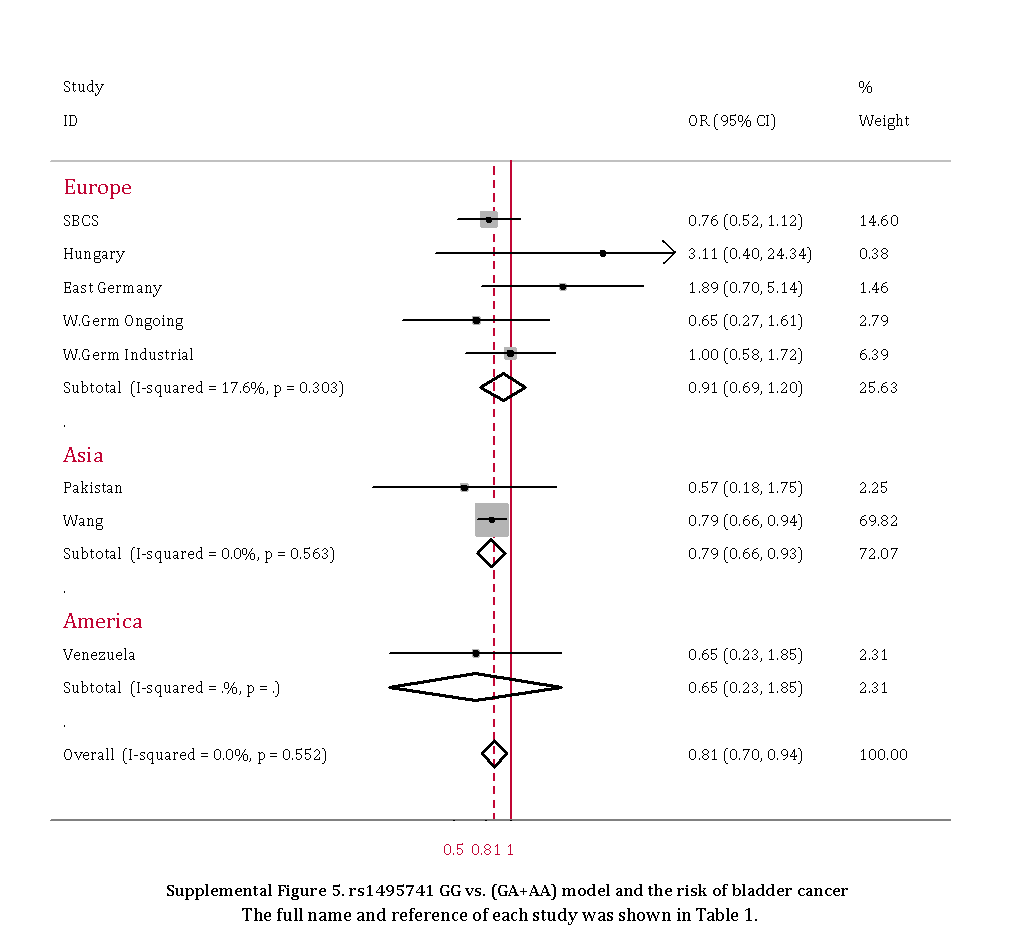


Supplemental Figure 6. rs1495741 (GG+GA) vs. AA model and the risk of bladder cancer


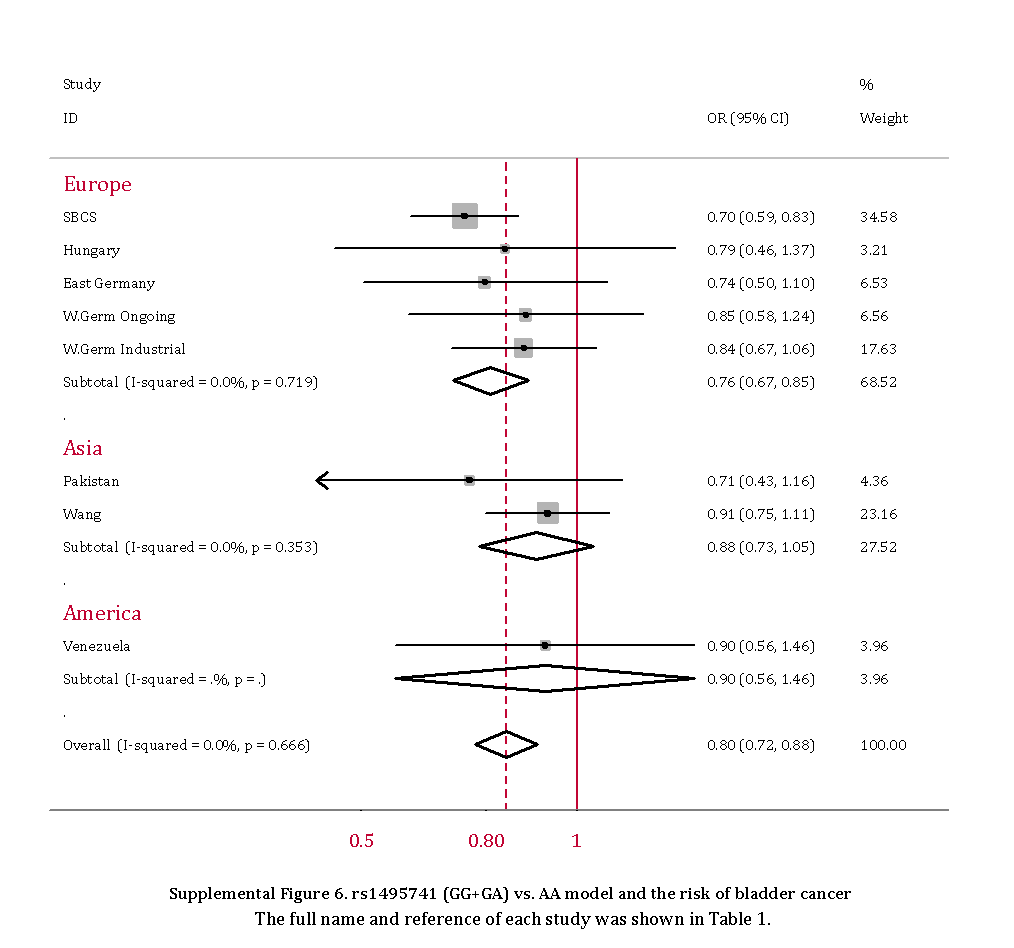


Supplemental Figure 7. Smoking and the risk of bladder cancer by region


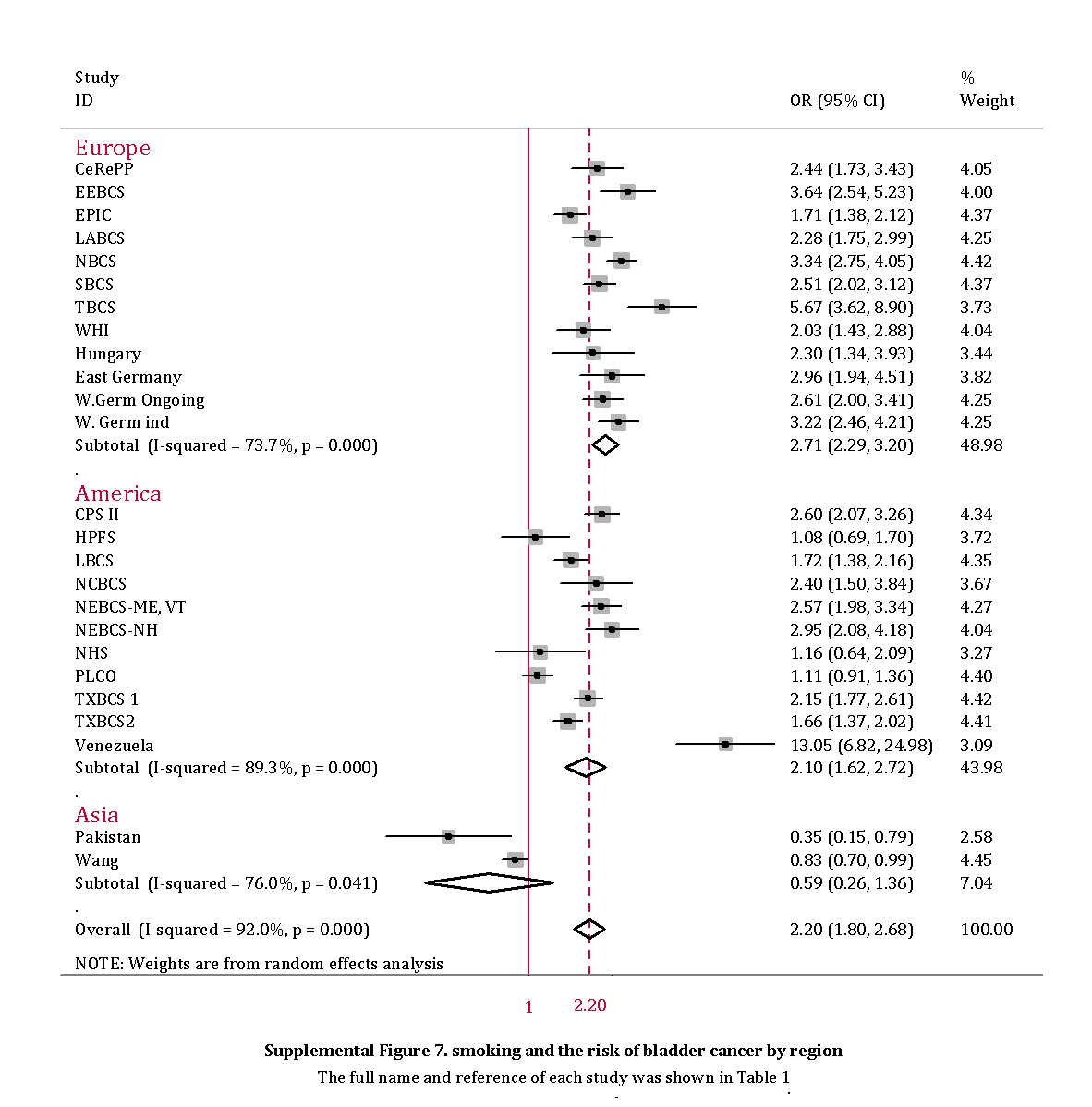


Supplemental Figure 8. Smoking and the risk of bladder cancer by design


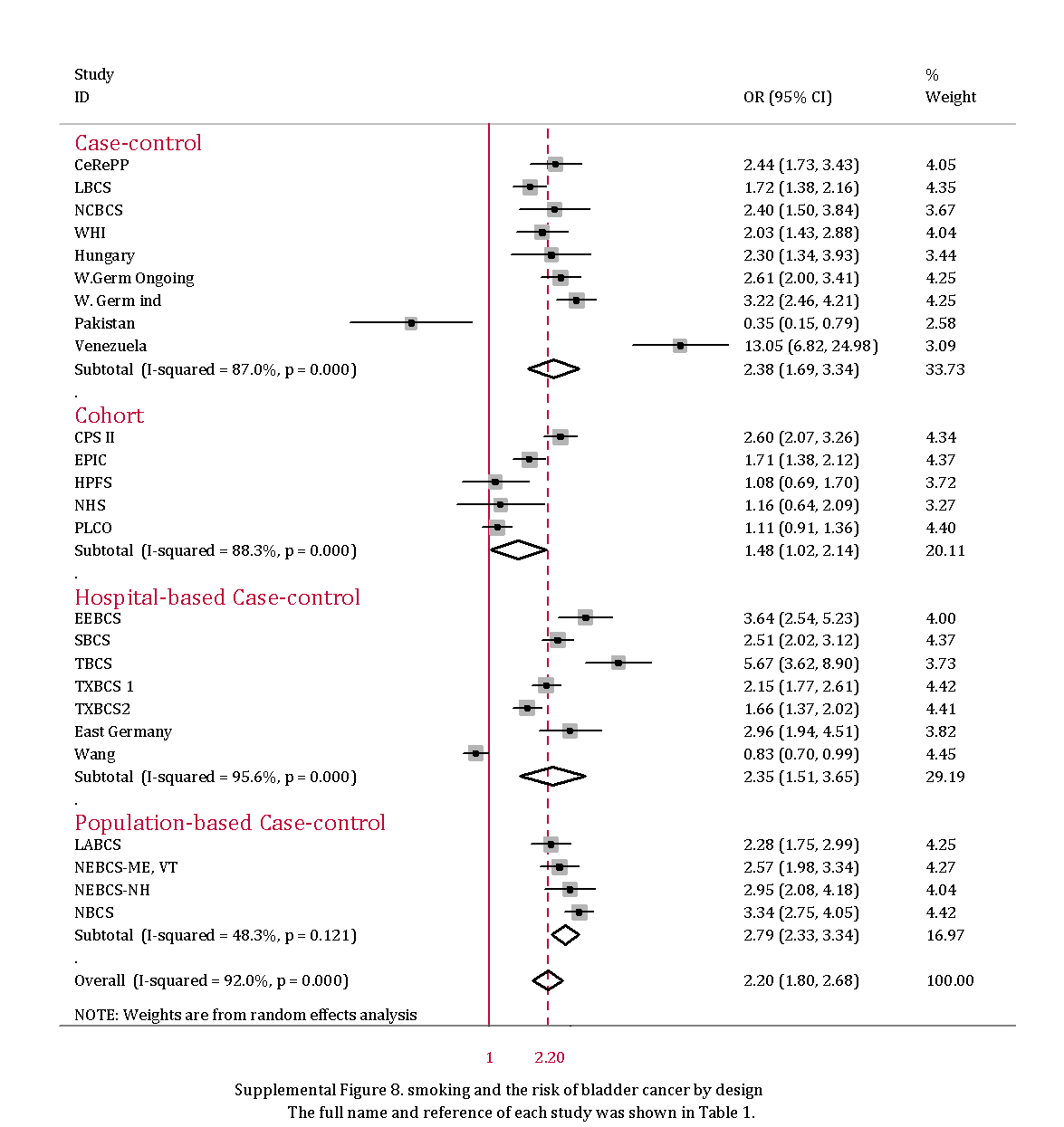


**Supplemental Table 1. Genotype distribution of 8 included articles**

| **Study** | **Region** | **Case** | **Control** | **Case** | | | | **Control** | | | |
| --- | --- | --- | --- | --- | --- | --- | --- | --- | --- | --- | --- |
|  |  |  |  | **AA** | **GA** | **GG** | **HWE** | **AA** | **GA** | **GG** | **HWE** |
| SBCS | Europe | 1106 | 1050 | 725 | 324 | 48 | 0.31 | 620 | 396 | 61 | 0.98 |
| Hungary | Europe | 226 | 76 | 166 | 85 | 12 | 0.97 | 38 | 27 | 1 | 0.29 |
| East Germany | Europe | 216 | 212 | 138 | 67 | 12 | 0.60 | 113 | 81 | 6 | 0.16 |
| W.Germ Ongoing | Europe | 318 | 173 | 200 | 107 | 11 | 0.77 | 102 | 62 | 9 | 0.99 |
| W.Germ Industrial | Europe | 443 | 964 | 274 | 149 | 20 | 1.00 | 522 | 341 | 41 | 0.29 |
| Pakistan | Asia | 106 | 61 | 70 | 29 | 4 | 0.90 | 135 | 75 | 15 | 0.59 |
| Venezuela | America | 112 | 190 | 52 | 45 | 5 | 0.48 | 92 | 84 | 14 | 0.68 |
| Wang | Asia | 1050 | 1403 | 222 | 540 | 288 | 0.57 | 275 | 675 | 453 | 0.71 |

The complete name of each study: Spanish Bladder Cancer study (SBCS),The Hungary Case-control series (Hungary), The Wittenberg Case-control series /Lutherstadt Wittenberg bladder cancer study (East Germany), West Germany-ongoing Case-control series (W. Germany Ongoing), West Germany–industrial burdened Case-control series (W. Germany industrial), The Pakistan Case-control series (Pakistan), The Venezuelan Case-control series (Venezuelan), Wang, Meilin, et al, 2014 (Wang).

HWE (Hardy–Weinberg equilibrium) was represented in P values.
